# Gluteal muscle contracture: diagnosis and management options

**DOI:** 10.1051/sicotj/2016036

**Published:** 2017-01-06

**Authors:** Saroj Rai, Chunqing Meng, Xiaohong Wang, Nabin Chaudhary, Shengyang Jin, Shuhua Yang, Hong Wang

**Affiliations:** 1 Department of Orthopedics, Wuhan Union Hospital, Tongji Medical College, Huazhong University of Science and Technology #1277 Jiefang Avenue 430022 Wuhan P.R. China; 2 Department of Radiology, Tongji Hospital, Tongji Medical College, Huazhong University of Science and Technology #1095 Jiefang Avenue 430030 Wuhan P.R. China

**Keywords:** Arthroscopy, Endoscopic surgery, Gluteal muscle contracture, Iliac hyper-dense line, Minimal invasive surgery

## Abstract

Gluteal muscle contracture (GMC), a debilitating disease, exists all over the globe but it is much more prevalent in China. Patients typically present with abduction and external rotation of the hip and are unable to bring both the knees together while squatting. Multiple etiologies have been postulated, the commonest being repeated intramuscular injection into the buttocks. The disease is diagnosed primarily by clinical features but radiological features are necessary for the exclusion of other pathological conditions. Non-operative treatment with physiotherapy can be tried before surgery is considered but it usually fails. Different surgical techniques have been described and claimed to have a better outcome of one over another but controversy still exists. Based on published literatures, the clinical outcome is exceptionally good in all established methods of surgery. However, endoscopic surgery is superior to conventional open surgery in terms of cosmetic outcome with fewer complications. Nevertheless, its use has been limited by lack of adequate knowledge, instrumentations, and some inherent limitations. Above all, post-operative rehabilitation plays a key role in better outcome, which however should be started gradually.

## Introduction

Gluteal muscle contracture (GMC), as the name suggests, is a clinical syndrome characterized by the contracture of gluteal muscles, iliotibial band (ITB), and related fascia, in severe cases hip external rotators and rarely hip joint capsule [1–3]. This debilitating disease was first described by Fernandez de Valderrama in 1969 [1]. Contracture leads to varying degrees of limitation of hip motion with hip deformity and even femoral head osteonecrosis [4]. Patients with GMC typically present with abducted and externally rotated hip and are unable to bring both knees together when squatting [5]. GMC occurs most commonly in children, usually bilateral, and the boys suffer more often than the girls [6].

Regarding the etiology, different possible hypotheses have been put forward, namely; idiopathic [7], genetic [2, 8, 9] or congenital [10, 11], and postnatal or acquired. Idiopathic GMC, a rare entity [12], may be associated with other diseases such as cerebral palsy [13], brain atrophy [14], poliomyelitis [2], and diseases with some unknown etiology [11]. On the other hand, acquired GMC is the commonest variety which has been proven to be associated with repeated intramuscular injections into the buttocks which in turn lead to fibrosis and contracture, otherwise known as “Injection-Contracture” [1, 4, 6, 15–18]. The younger the patients at the time of injection, the higher is the prevalence [19]. GMC persists all over the globe [3, 7, 16, 20–25] but it is much more prevalent in China with an overall childhood incidence rate of 1–2.5% [26–29], which is believed to be the result of the frequent use of benzyl alcohol as a diluent for intramuscular injection of antibiotics like penicillin [17, 30]. In Africa, intramuscular injections of quinine into the buttocks have been reported as the cause of gluteal muscle fibrosis [21, 31]. Other causes of acquired GMC may be injuries around the hip [32].

## Diagnosis

### Clinical features

GMC is diagnosed primarily by history and some important physical examinations (Table 1) [8]. Symptoms and signs vary depending on the severity of the disease. Abduction and external rotation along with a limited flexion and adduction of affected hip are the pathognomonic features of the disease [2, 33]. Patients are unable to bring their knees together when they squat (squatting test) or crouch [5]. Shen described this condition as “indeed some patients abduct the legs to such an extreme degree that they become straight-line – a posture that cannot be assumed by a normal person” [11]. There is always difficulty in crossing or overlapping the legs (cross sign) [4]. Active flexion test is positive [5]. Ober’s sign is positive [34]. In contrast to Ober’s sign which represents the contracture of iliotibial band and/or tensor fascia lata. Scully et al. (2015) described the term “reverse Ober’s sign” as a pathognomonic finding of gluteus maximus contracture, in which the progressive hip abduction occurs when extended and adducted hip is flexed to 90° or more [23].

**Table 1.** Clinical features of gluteal muscle contracture.

Symptoms	History of repeated intramuscular injections into the buttocks
	Abduction and external rotation with limited flexion and adduction of affected hip
	Unable to bring knees together during squatting, sits in frog-leg position
	Out-toeing gait/cannot walk in straight line
	Snapping sound while squatting
	Unable to cross or overlap legs
	Knee crepitus
	Anterior knee pain
Signs	Ober’s sign positive
	Active flexion test positive
	Reverse Ober’s sign positive
	Palpable snapping sound while squatting
	Pelvic tilt toward severe side
	Compensatory scoliosis
	Apparent leg length discrepancy (affected leg looks longer)
	Flattened or cone-shaped buttock
	Dimpling of skin in the buttock area

Other features include out-toeing gait, flattened and cone-shaped buttock, apparent leg length discrepancy, pelvic obliquity, and compensatory lumbar scoliosis [8, 35]. The leg appears longer on the involved side as there is pelvic obliquity due to continuous traction by contracture bands. While squatting, patients usually produce snapping sound as the fibrotic band glides over the greater trochanter, one may also palpate fibrotic band movement over greater trochanter [36]. Most of the patients have knee crepitus, most likely the consequence of chronic stress of rotational malalignment while they attempt to adjust the externally rotated knee [35]. Some patients may complain of anterior knee pain [37].

### Imaging

Although clinical findings are the most important in the diagnosis of GMC; radiological findings (Table 2), in some situations, could be helpful to support the diagnosis and rule out other pathological conditions [5, 8, 38]. Conditions such as acute muscle injury and associated fractures, denervation injury to the glutei, and other inflammatory conditions like iliopsoas abscess and tendinitis possibly mimic the clinical features of GMC. Radiological examination should be performed to rule out these conditions [10].

**Table 2.** Imaging modalities of gluteal muscle contracture.

	Features
Plain radiograph	1. Iliac hyper-dense line sign along the lateral iliac cortex in anteroposterior (AP) view
	2. Pelvic obliquity
	Other signs
	1. Increase in the neck shaft angle
	2. Reduction in center-edge angle
	3. External rotation of proximal femur
Magnetic resonance imaging (MRI)	*Primary features*
	1. Marked atrophy of gluteus maximus
	2. Intramuscular fibrous band
	*Secondary features*
	1. Medial retraction of the distal belly and tendon
	2. Posteromedial retraction of the iliotibial tract at attachment
	3. Depressed groove at the muscle-tendon junction
	4. External rotation of proximal femur
Computed tomography (CT) scan	1. Atrophy of gluteal muscles
	2. Calcification and necrosis of the injection site
	3. Curly band of fascia
	4. Widened gluteal muscle clearance
Ultrasonography (USG)	1. Thinning of involved muscles
	2. Hyperechoic bands within the muscle bundles suggest fibrosis

**Figure 1.**
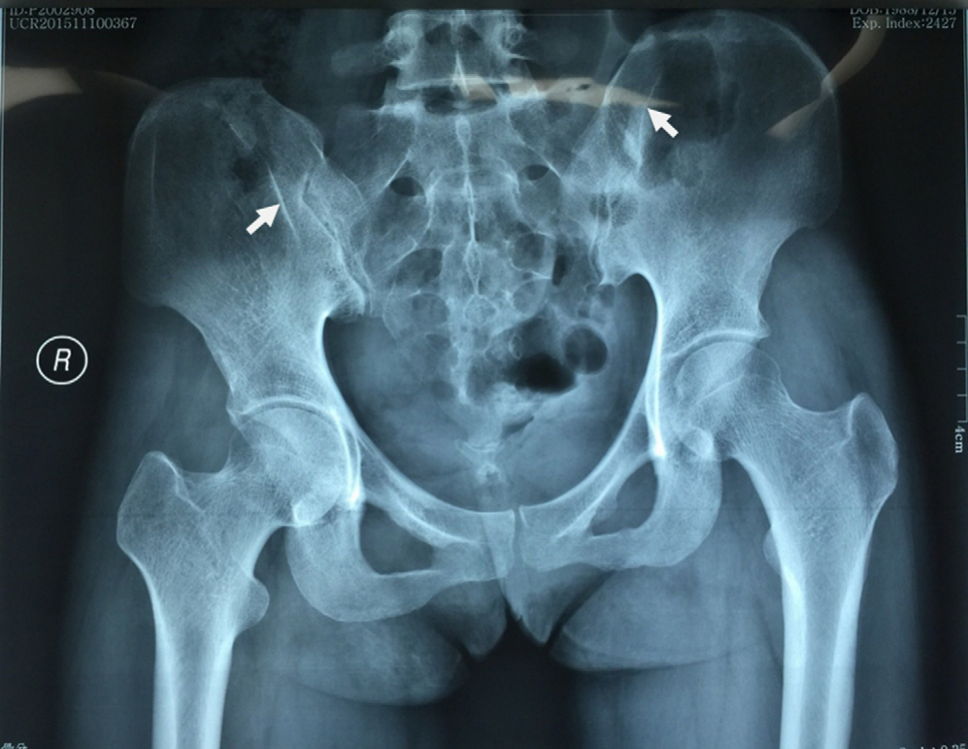
Anteroposterior radiograph of a patient with bilateral gluteal muscle contracture. The two arrowheads show iliac hyper-dense line over the bilateral posterior iliac spine with slight pelvic inclination toward the right.

A plain radiograph shows no significant changes in the early stage. On disease progression, the “iliac hyperdense line” (Figure 1) running parallel to the sacroiliac (SI) joint in the anteroposterior (AP) radiograph of pelvis is seen as a characteristic sign of the GMC, which perhaps results from the chronic tugging effect by contracted gluteus maximus on the lateral cortex of posterior ilium [10, 38, 39]. Other nonspecific signs are pelvic obliquity, a slight increase in neck shaft angle of the femur (coxa valga), and a reduction of the center-edge angle [6, 12, 39].

Magnetic resonance imaging (MRI), the modality of choice, shows marked atrophy of gluteus maximus in the presence of fibrotic bands, which appears as a low-intensity signal in all the sequences, which is most obvious in the fat-suppressed sequences. In advanced cases, medial retraction of the distal muscle belly and tendon of gluteus maximus along with the external rotation of the proximal femur and posteromedial retraction of iliotibial tract occurs. Also, a depressed groove appears at the muscle-tendon junction [5, 10]. Other imaging modalities include computed tomography (CT) scan and ultrasonography (USG) of the involved glutei. The CT scan may show gluteal muscle atrophy, calcification, and necrosis of the injection site, curly bands of fascia, and widened gluteal clearance [40]. The USG features are the thinning of involved glutei and presence of hyperechoic bands within the muscle bundles, signifying fibrosis [19].

## Classification system of gluteal muscle contracture

A number of classifications of GMC have been established by different authors in the past which mainly focused on the cosmetic aspect rather than the functional aspect of the disease [3, 11, 41]. Zhao et al. in 2009 and Ye et al. in 2012 proposed classifications of GMC that are fairly based on the clinical manifestations and anatomic changes and address the functional aspect of the disease [8, 35]. Zhao et al.’s classification consists of three levels and three types, whereas Ye et al.’s classification consists of three types. Both the classification systems do not seem to be much different from each other and both the classification systems are practically more reliable in understanding the disease pathology and useful in choosing the correct treatment options [22]. Zhao also recommended treatment options according to the severity of the disease as a non-operative or arthroscopic treatment for level I disease, an operative treatment especially an arthroscopic treatment for level II disease, and an operative treatment under direct vision with a conventional incision for level III disease [8].

## Treatment options

**Figure 2.**
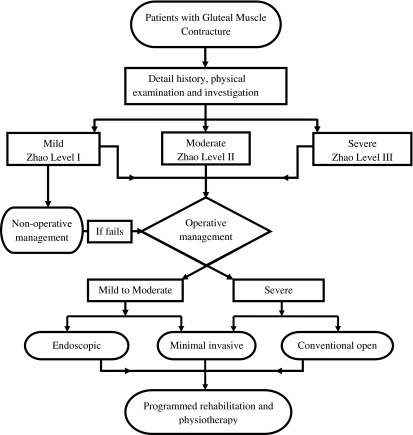
Flowchart of management options for gluteal muscle contracture [8].

The treatment options have been well illustrated in the flowchart (Figure 2). It includes non-operative treatment, different operative treatments, and programmed rehabilitation and physiotherapy.

### Non-operative treatment

Non-operative treatment is indicated only in mild cases or is recommended for those patients who are not eligible for surgery or are waiting for surgery. It includes massage, physiotherapy, shortwave diathermy, and active and passive stretching exercises [8]. However, the effectiveness of non-operative treatment is higher in children than adolescent and significantly superior in Zhao level I diseases than in level II and level III but it is still lower than expected [8, 42]. It is said that once the contracture is established the non-operative treatment has no role [1, 35, 43].

### Operative treatment

Operative treatment is the gold standard method of treatment for all the established cases of GMC [43, 44]. Different operative methods have been introduced, which include conventional open release, endoscopic release, and minimally invasive release method. Surgery can be performed under general, lumbar spinal, or epidural anesthesia according to the availability of experts and patient’s tolerability, but some authors prefer epidural anesthesia as having the least effect on the patient’s general health [35]. However, these treatment methods have their own merits and demerits (Table 3). Meticulous care should be taken to minimize complications, especially avoiding sciatic nerve injury.

**Table 3.** Literature review of surgical options of gluteal muscle contracture and therapeutic outcome.

References	Study design	Sample size	Age	Treatment given	Treatment outcome	Complications/Recurrence
Gao 1988 [12]	Retrospective	27	8.5 years (3–14)	Open	Good result in all	One had acute hematoma
						Two patients had restricted motion
He et al. 2003 [42]	Retrospective	187	9 years (3–27)	Open	Good/excellent result = 97%	Cicatricial band formation = 62, hematoma formation = 6, wound infection = 3, wound dehiscence = 1
Ekure 2006 [21]	Retrospective	28	5.6 years (9–12)	Open	Excellent in all	Deep sepsis = 2
						Temporary sciatic nerve palsy = 1
Zhang et al. 2007 [32]	Retrospective	2518	5–30 years	Open	Excellent = 2260	Infection = 4, hematoma = 5, bruising = 15, temporary sciatic nerve injury = 3, LFCN injury = 8, instability = 3, permanent sciatic nerve injury = 6
					Good = 252	Recurrence = 4
Zhao et al. 2009 [8]	Retrospective	129	7.4 years (4–17)	Open	83.7% excellent result	Complications after operative management only appeared in level II and III patients, which included hypertrophic scar (II = 16, III = 48 [some severe cases exceeded 7 mm]), hematoma (III = 4), infection (II = 1; III = 1), and wound dehiscence (III = 1)
Liu et al. 2011 [4]	Retrospective	428	8 years (5–15)	Open	Excellent = 400	Six patients under 5 years had fair result due to poor compliance; 16 patients had unsteadiness in walking
					Good = 22	
Liu et al. 2009 [29]	Retrospective	108	23.7 years (18–40)	Arthroscopic	Adduction	None
					From 10.4° to 45.3°	
					Flexion	
					From 44.8° to 110.2°	
					Out-toe gaits correction with different degrees	
Fu et al. 2011 [44]	Comparative	Open 50	8.9 years (6–19)	Open	47/50 Good/excellent, 32/50 cosmetic satisfaction, 47/50 functional satisfaction	Recurrence = 1
		Endoscopic 52	9.2 years (5–20)	Arthroscopic	46/52 Good/excellent, 48/52 cosmetic satisfaction, 46/52 functional satisfaction	Recurrence = 1
Liu et al. 2013 [48]	Retrospective	358	19.7 years (14–41)	Arthroscopic	303 Excellent, 13 good	None
Ye et al. 2012 [35]	Retrospective	1059	23 years (8–43)	Minimal invasive	Excellent in all	Acute painful hematoma = 3, minimal complications like pain, swelling, shuffling gait, muscular weakness around hip joint, and keloid formation

#### Conventional open surgery

**Figure 3.**
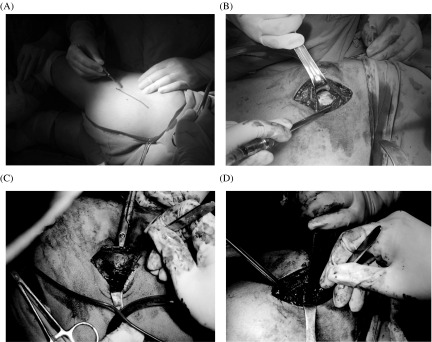
Conventional open gluteal muscle contracture release. (A) The patient was positioned laterally with hip in neutral, a longitudinal skin incision line was drawn over the left buttock; (B) a skin incision was made along the marking line, a fibrotic contracture band appeared as a silvery white structure over the greater trochanter; and (C) and (D) show the division of contracture bands under direct vision, starting from superficial to deeper structures.

The conventional open release of GMC has a very old history. It is indicated in all established cases but it is highly recommended in severe cases because wide incision provides appropriate exposure allowing the division of fibrotic bands under direct vision (Figure 3). It involves variable length and shape of skin incision (5–12 cm) usually in the lateral position over buttock and greater trochanter according to the surgeon’s preferences and experience, followed by the division of contracture band [1]. Different shapes of skin incisions include transverse straight, curved, longitudinal straight, and “S”-shaped incision, however, an “S”-shaped incision over the greater trochanter is most efficient in terms of clear exposure, less tissue damage, high safety rate, excellent results, and low recurrence rate [45]. The division of contracture band is performed in a sequential manner according to the anatomy of the muscle group involvement (ITB, gluteus maximus, gluteus medius, gluteus minimus, other external rotators, and even joint capsule) starting from superficial to deeper structures until all the signs and symptoms completely disappear intra-operatively. The intra-operative examination includes adduction, flexion, internal rotation, Ober’s sign, cross leg, and palpable click. Any residual deformity may lead to failure of surgery. Some surgeons advocate Z-plasty to release contracture bands having a better outcome [1, 45–47].

#### Endoscopic release surgery

**Figure 4.**
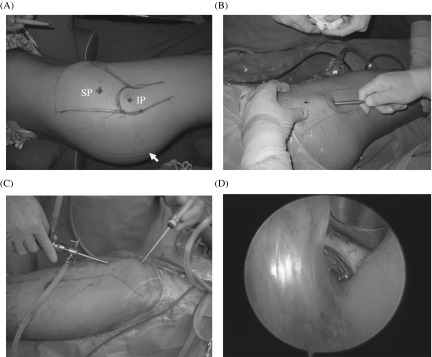
Endoscopic release of gluteal muscle contracture using two portals technique. (A) In neutral lateral position of the hip, important anatomical landmarks were drawn. IP represents inferior portal or viewing portal (3 cm distal to superior border of greater trochanter) whereas SP represents superior portal (5 cm proximal to IP) which is working portal; and an arrow points the course of sciatic nerve; (B) surgeon created an artificial working space; (C) represents endoscopic release of gluteal muscle contracture in lateral position and; (D) shows silvery white contracture bands.

The introduction of arthroscopy-guided radiofrequency ablation of GMC was first reported by Liu et al. in 2009 [29]. It is mainly indicated in Zhao level I and II, and very cautiously in level III [8, 44]. The procedure involves the marking of all important anatomical landmarks like greater trochanter, anterior and posterior borders of contracted glutei, and course of the sciatic nerve in the lateral position (Figure 4A) [29, 44]. Usually, two (Figure 4B) or three portals are made according to variation in the location and depths of GMC groups. After the introduction of arthroscope in the artificial space created around the greater trochanter, a silvery white band of contracture is divided using a radiofrequency ablation device starting from superficial to deeper structures (Figures 4C and 4D). There is always a chance of bleeding from muscles, which may be prevented by the prophylactic use of adrenalin (1 mg in 3 L) in a continuous flow of normal saline and any other visible bleeders are also coagulated instantly [29]. Intra-operatively, the confirmation of complete release should be made using the same test as in conventional open surgery.

Advantages of this technique are small surgical wound, short operative time, earlier rehabilitation, and return to functional activities and minimal complications. However, the precise selection of patients is critical for the optimum outcome of surgery and one must not forget its innate weakness.

#### New minimally invasive surgery

New minimally invasive open release of GMC has been introduced by Ye et al. (2012). This procedure can be considered in all cases of GMC. Preoperative physical examination confirms the extent of disease better. While performing this procedure the surgeon must have meticulous knowledge and skill regarding anatomical landmarks and operative procedure, as a complete division of contracture bands is the mainstay of the surgery. The surgeon performs this procedure using small incisions in different anatomical points in the supine position around the greater trochanter and utilizes a specially designed scalpel to divide contracture bands [35]. Confirmation of the complete division can be made using the same technique as mentioned above.

The advantage of this procedure over others is that it is simple and easy to perform, has small surgical wound and cosmetic benefits, short operative time, and it is effective even when deeper structures are involved [35]. Although the procedure seems simple and easy to perform, the surgeon should never forget that it is a blind procedure and has full chances of complications.

## Post-surgical treatment and rehabilitation

**Figure 5.**
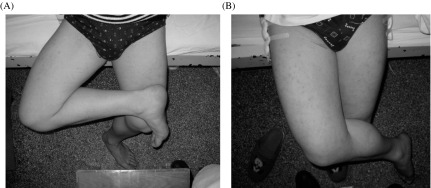
Pre-operative vs. post-operative photograph of a patient with bilateral GMC who underwent endoscopic release using the two-portal technique. (A) The patient demonstrated an abducted and external rotation contracture of the right hip preoperatively where the patient was unable to cross his leg; whereas (B) immediate post-operative photograph: the patient was able to cross the legs.

Post-operative rehabilitation is crucial for rapid recovery and optimum clinical outcome [4]. The post-operative treatment starts immediately after the surgery. This includes adequate vitals’ monitoring, pain and anxiety management, and passive and active stretching exercises. Generally, no immobilization or traction is necessary [7]. Hematoma formation is the most common immediate complication after surgical release of contracture, which may be prevented by the adequate wound and drainage care. The patients are usually encouraged to lie down on lateral position, which ensures sufficient wound compression on one side by their body weight while on the other side a 2 kg ice bag is placed and every 1–2 h the position is switched in case of bilateral contracture release [29, 45]. The rehabilitation protocol is similar for all procedures; however, the initiation time may vary as a minimally invasive technique has a small skin incision, which usually starts after the drainage tube’s removal within 24–48 h. The patient is instructed to do functional exercise after the elimination of post-surgical pain or after the drainage tube is removed [45]. Exercise is started with passive and active flexion of the knee and hip, then the patient is allowed to walk and gradually perform other exercises which include crossing legs (Figure 5), walking straight, and crouching with closed knees [35, 44, 45].

In the patient with an apparent leg length discrepancy, both pelvic lift exercise and skin traction are recommended to correct the discrepancy [4, 47]. An early vigorous exercise may induce hematoma, so it is avoided until the wound is fully healed, usually for three weeks [35]. The rehabilitation is continued for at least six months [35]. The patient is discharged from the hospital once they can walk freely without any walking aids after suture removal [45].

## Discussion

Repeated intragluteal injections of antibiotics and antimalarial agents are found to be the major causes of GMC which is still in practice, especially in developing countries. Two rationales have been explained, first being repeated intramuscular injections of antibiotics and its diluents causing direct effect on healthy muscles, and second being the physical injury caused by a large volume of fluid delivered with repeated injections, both causing muscle inflammation followed by fibrosis. Patients typically present with abduction and external rotation along with a limited flexion and adduction of the affected hip, a pathognomonic feature of GMC. Disease diagnosis is mostly made by clinical features; however, radiological examination should be considered to rule out acute muscle injury and associated fractures, denervation injury of glutei, and other inflammatory conditions like iliopsoas abscess and tendinitis [10]. However, an anteroposterior radiograph of the pelvis may be normal in the initial stage of the disease, except some degree of pelvic inclination and external rotation of the hip but in a longstanding disease, the iliac hyper-dense line may be evident (Figure 1). The MRI shows atrophy of involved muscles and fibrotic bands, especially in fat-suppressed sequences. Other imaging modalities like CT scan and USG may be helpful in disease diagnosis and exclusion of any other pathology. Whatever the etiology, definitive diagnosis of the disease is crucial for appropriate treatment.

Despite the fact that non-operative treatment of GMC has a poor outcome, it can be tried before any surgery is considered or if the patient compliance is poor [1]. Liu et al. (2011) did not advise surgery in children aged under five years as they are unable to follow strict post-operative rehabilitation [4]. Zhao et al. (2009) reported that non-operative treatment was effective only in 38% out of 49 patients regardless of the very strict rehabilitation protocol [8]. A similar result was reported by He et al. (2003), in their case series; only 39% of patients had good to excellent result with physiotherapy [42]. Although, only these data are not sufficient to conclude that the non-operative treatment has no/less role, indeed provides some imperative evidence that the non-operative treatment is not that effective even in Zhao level I.

In established cases of GMC, surgical release is the treatment of choice, however, the choice of surgery is truly dependent on the correct classification of disease and the availability of experts and advanced tools. Open surgical release is being performed since decades with excellent result; however, multiple authors have reported that the large surgical trauma significantly augments post-operative complications like acute painful hematoma, bruising, wound infection, hypertrophic scar formation, wound dehiscence, and neurovascular injury. Thus, delaying rehabilitation might lead to severe morbidity and cosmetic dissatisfaction to the patients [35]. Reports suggest that the patient who underwent Z-lengthening of contracture bands especially ITB requires prolonged rehabilitation to achieve full range of active hip motion [11, 35]. Some degrees of Trendelenburg gait post-operatively may be evident in some patients due to the extensive release of hip abductors especially the gluteus medius [8].

He et al. (2003) performed 187 open surgical release and found 97% good to excellent results; however, 62 patients had hypertrophic scar formation, six acute hematoma formation, three wound infection, and one wound dehiscence [42]. Similarly, in a study performed by Zhang et al. (2007) with a large volume of cases (*n* = 2518), they encountered six cases of permanent sciatic nerve injury and four cases of recurrence. Other minor complications were four wound infection, five hematoma, 15 bruising, three temporary sciatic nerve injury, eight lateral femoral cutaneous nerve of thigh injury, and three hip joint instability. Hip joint instability recovered after regular exercise [32]. Zhao et al. (2009) reported in their case series of 129 patients with open release, 62 patients had a hypertrophic scar, four hematoma, two infection, and one wound dehiscence [8]. Ekure (2006) revealed intramuscular injection of quinine as the major cause of GMC in Africa. He reported excellent result in all the cases in terms of hip range of motion, however, two cases had deep infection and one had sciatic nerve injury [21]. Al Bayati et al. (2015) reported seven cases of GMC in Iraq, where the conventional open release was performed. The patients were followed up for two months to 12 months and the results were excellent in all the cases without any known complications [22]. Scully et al. (2015) reported four cases of injection-induced GMC in the United States of America in children who were previously adopted from East Europe and China; the authors reported that the entire patients had high satisfaction as they could participate in sports activities in the school; however, one had infected hematoma requiring interventions and antibiotics treatment [23].

These well-known complications of GMC after conventional open surgery created a negative impact on the patients’ functional as well as cosmetic satisfaction, especially in youngsters, thus it has become a great concern for orthopedic surgeons to seek other surgical techniques.

Endoscopic release of GMC is the new and emerging technique, only limited numbers of studies have been performed, however, the outcome is comparable to or even better than the open conventional surgery [29, 44]. Liu et al. (2009) assumed that arthroscopic release of GMC would avoid the extensive surgical trauma caused by precise and selective contracture releases in an extremely controlled way, thus providing acceptable outcome and minimizing complications related to open surgery [29]. They reported excellent result in terms of range of motion (flexion and extension) with minimal complications. Moreover, Fu et al. (2011) compared endoscopic release with conventional open surgical release; they also reported significant superior result with endoscopic group in terms of small surgical trauma, less post-surgical pain, early off-bed activity time, short hospital stay, and cosmetic satisfaction, but there were no statistical differences in the duration of surgery, complications, clinical outcome, and 1-year recurrence rate. Four patients in the endoscopic group having large GMC (Zhao level III) had a disappointing outcome with arthroscopy, and the treatment was converted to open release, which indicates that there are always some innate limitations of the endoscopic technique, so a precise selection of patient is utmost for successful outcome [44].

Although this technique has fewer complications with the comparable clinical outcome, it is highly specialized, hence a surgeon must have immense knowledge about instrumentations and procedure. Meticulous preoperative clinical examinations and diagnosis are crucial in order to prevent complications and recurrence. An arthroscope may not be that effective to visualize deeper structures like gluteus medius, gluteus minimus, piriformis muscle, and joint capsule. In a similar way, a large amount of normal saline used to create operative field may have a negative impact on healthy muscles [35].

A new minimally invasive open release technique has been described by Ye et al. (2012) [35]. They performed surgery in a large number of patients (*n* = 1059), followed up for six months to five years (mean 2.5 years), and reported an excellent outcome according to their evaluation criteria, with a mean of 2.6 weeks for Ye et al. type A, 3.2 weeks for type B, 3.5 weeks for type C1, and 11.5 weeks for type C2 [35]. Though it was not without complications, three patients had acute rupture of a branch of the circumflex femoral artery at the neck of femur, which was managed successfully with a small incision [35]. However, this technique seems to be easier with fewer complications, even though the technical difficulties and limitations have not been described by the author properly. No other publications regarding this technique have been released yet. Since this procedure is performed with the blind eye with small incisions, the chance of incomplete release is possibly high with possible neurovascular injuries. These anatomical landmarks indeed differ in different age groups or height. Adequate knowledge and clinical skills are necessary for successful outcome.

## Conclusion

Despite various complications related to the large surgical incision, multiple studies signify that the open release is effective in all levels of disease. Minimally invasive treatment methods have a superior result with high cosmetic satisfaction and fewer complications especially in youngsters, so the surgeon must think about choosing an arthroscopic technique. However, a thorough clinical and radiological examination is crucial to make a correct treatment plan. The endoscopic release can be performed successfully in Zhao level I and II, and very cautiously in level III, but one should never forget the inherent limitations of arthroscopy. Open surgery should always be reserved for big and complicated gluteal muscle contractures, so we must not devalue its option just because of the surgeon’s pursuit of any other minimal invasive choice.

## Conflict of interest

The authors declare that there is no conflict of interest with any financial organization, corporation, or individual that can inappropriately influence this work.
